# Obesity and Its Treatment

**Published:** 1904-05-21

**Authors:** 


### Hospital Clinics.

### OBESITY AND ITS TREATMENT.

Though general excess of adipose tissue in an individual is not commonly regarded as a disease, there is no especial reason why it should not be considered so. The accumulation of an undue amount of fat is indeed a pathological process induced by faulty habits of life, and it would perhaps be advantageous if this fact were to receive wider acceptance. The condition is due to a loss of balance between intake and output; either too much food is taken or too little is oxidised. The exact nature of the want of balance may usually be found, according to Dr. Leonard Williams,[1] if inquiry is made as to ingested materials, their quantity and quality, and into the facilities which the patient's mode of life affords to the normal oxidising processes. He believes that the custom of eating to excess not infrequently has its origin in the days of youth and athletics. Out-door games and exercises during the years of bodily development lead to the taking of copious meals, a habit which is liable to remain after the more serious business of life has set its limits to the individual's opportunities for physical activity. Though nothing more than a habit, the obvious remedy for over-eating is by no means always easy to apply. Another common cause of the ingestion of too much food is the practice of drinking with meals. The fluid not only increases the appetite, but it enables the food to pass more rapidly out of the stomach than it would otherwise do, and so makes it possible to eat more. The best plan to adopt is to drink the whole amount necessary about half an hour before food or else after all the solids have been eaten. Dr. Williams considers that insufficient mastication also is a frequent cause of taking too much food, and states that if every mouthful be masticated until it is both fluid and tasteless the amount of food which it is possible to take at a meal is reduced by at least one half. Another cause of over-eating is to be found in too frequent meals, and eating between the fixed meal times.

The dietetic fault may lie with the quality as well as the quantity of food, the excess revealing itself in the relative quantities of fat-forming and fat-saving principles. It is upon the recognition of this fact that the various "systems" for dietetic treatment are based. In connection with these systems it is necessary to remember two things. The first is that if the restrictions in any one of them are strictly adhered to the patient is not only being starved of fats and carbohydrates, but he is also being severely limited in the whole amount of food taken, and the treatment may produce serious weakness. The other is that such food as is permitted being nitrogenous and drawn almost entirely from the animal kingdom, there is grave danger of trouble arising in the excretory organs; and Dr. Williams states that he has known more than one case in which granular nephritis was attributable to a too rigorous application of the dietetic treatment.

The other main causative factor of obesity, namely, the inadequate oxidation of the food taken, is most commonly due to want of exercise, and it should be remembered that the effect of muscular contraction in oxidising superfluous adipose tissue is not only general but local. The deposit takes place in the abdominal wall and intra-abdominally, mainly because in the majority of people the abdominal muscles are very little exercised. In people whose work necessitates the vigorous use of these muscles, boatmen for example, the aldermanic figure is an exception. Not only bodily exercise but mental activity also has an effect on the production of excessive fat.

The neglect, which is so general, of the proper care of the skin is another factor in the production of obesity. Hot baths and habitual over-clothing are responsible for a great loss of the contractile power of the skin, and this is a contributing factor in the deposition of fat on the same principle by which excess of abdominal fat is due to lack of exercise of the muscles of the abdomen. The fear of draughts and of fresh air in general also plays its part.

With regard to treatment, this will be largely indicated by an estimation of the causative factors in each individual instance, but nowhere can the essential details be so readily or effectively enforced as at the leading health resorts. For at these places the meals, the hours, the whole life of the patient can be regulated. The change of scene and circumstance promotes mental activity, regulated bodily exercises, both natural and artificial, can be taken, the excretory organs can be brought into wholesome activity by means of baths and "taking the waters," and the whole regimen can be arranged for safely diminishing the tendency to fat production, while entailing the least possible amount of discomfort or feeling of hardship upon the patient. Another method of treating obesity is that by thyroid extract. Like some of the drastic dietetic systems, it is certainly efficacious in most instances, but it has this further resemblance to them, that its use is by no means unattended by danger. As Dr. Williams points out, the restlessness and tachycardia to which it is liable to give rise often persist for long periods after the drug is withheld, and unless its exhibition is carefully supervised and associated with the other details of management and hygiene, though it may reduce obesity, it will probably give rise to other troubles in comparison with which obesity might well be regrded as an ornament.

[1] Practitioner, May.

